# Bone Transport for Treatment of Traumatic Composite Tibial Bone and Soft Tissue Defects: Any Specific Needs besides the Ilizarov Technique?

**DOI:** 10.1155/2020/2716547

**Published:** 2020-02-24

**Authors:** Runguang Li, Guozheng Zhu, Chaojie Chen, Yirong Chen, Gaohong Ren

**Affiliations:** ^1^Department of Orthopedics, The Third Affiliated Hospital of Southern Medical University, Guangzhou 510610, China; ^2^Orthopaedic Hospital of Guangdong Province, Guangzhou 510610, China; ^3^Academy of Orthopaedics of Guangdong Province, Guangzhou 510610, China; ^4^Department of Orthopaedics, Nanfang Hospital, Southern Medical University, Guangzhou 510515, China; ^5^Guangdong Provincial Key Laboratory of Bone and Cartilage Regenerative Medicine, Nanfang Hospital, Southern Medical University, Guangzhou 510515, China; ^6^Department of Orthopedics, Panyu Hospital of Chinese Medicine, Guangzhou 511400, China

## Abstract

**Objective:**

To evaluate the surgical efficacy of bone transport (Ilizarov technique) plus “shortening-lengthening,” “flap surgery,” and “open bone transport” as individualized treatments for traumatic composite tibial bone and soft tissue defects.

**Methods:**

We retrospectively analyzed sixty-eight cases (mean age: 35.69 years, (range, 16–65)) treated from July 2014 to June 2017, including 29 middle, 18 distal, and 21 proximal tibial bone defects (4–18 cm, mean: 7.97 cm) with soft tissue defects (2.5 cm × 4.0 cm to 30.0 cm × 35.0 cm after debridement). We adopted the bone transport external fixator to fix the fracture after debriding the defect parts. In the meantime, we adopted the “shortening-lengthening technique,” “flap surgery,” and “open bone transport” as individualized treatment based on the location, range, and severity of the composite tibial bone and soft tissue defects. Postoperative follow-up was carried out. Surgical efficacy was assessed based on (1) wound healing; (2) bone defect healing rate; (3) external fixation time and index; (4) incidence/recurrence of deep infection; (5) postoperative complications; and (6) Association for the Study and Application of the Methods of Ilizarov (ASAMI) score.

**Results:**

The mean duration from injury to reconstruction was 22 days (4–80 d), and the mean postoperative follow-up period was 30.8 months (18–54 m). After the repair and reconstruction, 2 open bone transport patients required infected bone removal first before continuing the bone transport treatment. No deep infection (osteomyelitis) occurred or recurred in the remaining patients, and no secondary debridement was required. Some patients had complications after surgery. All the postoperative complications, including flap venous crisis, nail channel reaction, bone nonunion, mechanical axis deviation, and refracture, were improved or alleviated. External fixation time was 12.5 ± 3.41 months, and the index was 1.63 ± 0.44. According to the ASAMI score, 76.47% of the outcomes were good/excellent.

**Conclusion:**

The Ilizarov technique yields satisfactory efficacy for composite tibial bone and soft tissue defects when combined with “shortening-lengthening technique,” “flap surgery,” and “open bone transport” with appropriate individualized treatment strategies.

## 1. Introduction

Stable fixation of fractures, early coverage of wounds, and effective prevention and treatment of infection are the fundamental management principles for severe open tibial fractures [1, 2]. Early coverage of the wound refers to covering the wound as soon as possible after the necrotic tissue is cleaned. It is difficult to estimate the vitality of the damaged tissue, especially for firearm injuries in the war, which brings more difficulties for treatment [3]. The importance of early, multiple, and thorough debridement is widely accepted. However, larger tibial and soft tissue defects are caused by debridement in some cases [4], which significantly increases the difficulty of subsequent repair and reconstruction. Nonetheless, great progress has been achieved in the treatment of severe composite tibial bone and soft tissue defects. The following methods can be combined or adopted alone in clinical practice: flap surgery, free vascularized bone graft (fibula [5, 6], ilium [7]), bone transport (Ilizarov technique) [8–22], Masquelet technique [11], simple bone graft after wound closure [23], and Papineau technique for open bone graft [24]. Among them, bone transport has become the primary method to treat large bone defects owing its success to improved external fixators, more precise surgical procedures, and new insights into “autologous bone tissue engineering technology” and “regenerative medicine.”

Discomfort exists at all stages of the bone transport process, which significantly impairs quality of life [10]. Reducing the time of the patients with an external fixator and improving the quality of their life, maximizing the advantages of the Ilizarov technique, and choosing the appropriate individualized treatment are critical. Multiple techniques should be combined and individualized treatment applied during each treatment step, such as early debridement, vacuum sealing drainage, and antibiotic carrier technology. During repair and reconstruction, the shortening-lengthening technique, flap surgery, and open bone transport can be selected in addition to bone transport according to the individual condition of the patient. In this investigation, clinical data from 68 cases of traumatic composite tibial bone and soft tissue defects undergoing bone transport from July 2014 to June 2017 were collected and retrospectively analyzed to evaluate the surgical efficacies of these individualized treatments.

## 2. Materials and Methods

### 2.1. Inclusion and Exclusion Criteria

Inclusion criteria were as follows: (1) age 16–65 years, (2) tibial defect >4 cm after traumatic debridement accompanied by soft tissue defects (i.e., the wound could not be directly sutured after debridement), and (3) an external fixator could be placed in the proximal lower extremity, distal lower extremity, or foot, and normal bone segments were available for osteotomy. Exclusion criteria were as follows: (1) loss to follow-up, (2) external fixator was changed to internal fixator, (3) no possibility of preserving the lower extremity due to local defects, and (4) patients unsuitable or unable to tolerate surgery.

### 2.2. Patients and Methods

According to the inclusion and exclusion criteria, 68 cases were retrospectively assessed (42 males and 26 females, age 16–65 years, average age: 35.69 years). Defects were located on the left side in 30 cases and the right side in 38 cases. Forty-four cases were traffic accident injuries, 14 falling injuries, and 10 crush injuries. Among them, 35 cases were transferred to our hospital after treatment in a local hospital. Injury site was the middle tibial bone in 29 cases, distal in 18 cases, and proximal in 21 cases. The length of the tibial bone defect ranged from 4 to 18 cm (7.97 cm on average), and soft tissue defects ranged in area from 2.5 cm × 4.0 cm to 30.0 cm × 35.0 cm after thorough debridement. Eighteen cases were complicated by ipsilateral extremity fractures, 20 cases with fractures at other sites, and 12 cases with other systemic injuries. The time from injury to repair and reconstruction ranged from 4 to 80 days (22 days on average).

### 2.3. Surgical Procedures

Broad-spectrum antibiotics were applied at the early stage of therapy. Individualized treatment was designed according to the severity of the injury and staged surgery was performed. Emergency management was conducted by following the principle of “damage control.” Wound debridement was performed before repair and reconstruction as previously described [25–27]. After debridement, VSD or KCI vacuum sponge was temporarily utilized to cover the wound. Each cycle of VSD or KCI was maintained for 4–7 days. Patients with severe contamination, unclear margins of necrotic tissues, or wound surface infection received repeated or enlarged debridement.

According to the fracture site and severity of defects, a unilateral or circular external fixator or unilateral-circular external fixator (OrthoFix Medical Inc., Italy or Tianjin Xinzhong Medical Devices Co., Ltd., China) was selected. The monoaxial fixator (LRS fixator) is a stable, easy-to-use, and very handy device; as a result, it is preferable to use [28]. The fixation method and needle insertion paths were based on the approaches of Nayagam [29]. Bilateral ends of the bone defects were repaired and leveled. Unhealthy tissues, such as those with inflammatory granulation, sinus tract, and unstable scar surrounding the bone defects, were thoroughly eliminated. The fibula was cut off at the middle or upper segments when necessary, and the lower leg was shortened. A proximal or distal tibial osteotomy was performed simultaneously or during the subsequent treatment stages. In addition to bone transport, we also applied the shortening-lengthening technique, flap surgery, and (or) open bone transport for wound repair. Among 68 enrolled patients, the wound area was reduced through limb shortening in 47 cases, while the remaining 21 cases did not undergo limb shortening. Specifically, 11 cases received subsequent direct suture, 25 cases received wound suture by local flap transfer, 19 cases underwent free flap transfer to repair the wound, and 13 cases received wound repair via bone transport.

Postoperative management was conducted according to the classic Ilizarov method [21], with bone transport performed at 0.5–1 mm/day, 2–4 times per day starting one week postoperative. An X-ray examination was conducted on a regular basis. The speed of bone transport was adjusted according to the new callus. For patients treated with the shortening-lengthening technique, limb shortening was performed gradually according to the specific conditions after surgery to reduce the tension on soft tissues. Bone transport was completed toward bilateral ends, followed by lengthening to restore the normal span of the tibia. For patients undergoing flap surgery, careful observation and necessary braking were required. Anti-infection, antispasm, and anticoagulation therapies, as well as conventional microsurgical methods, were applied to preserve the blood supply of the flap. For those receiving open bone transport, it was necessary to provide more aggressive nursing of the wound. Specifically, the dressing was changed every 2-3 days, and Vaseline gauze or antibacterial gauze was used to keep the wound dry and clean after pulling until the wound was healed. Postoperative follow-up was conducted on a regular basis. According to the problems identified during postoperative follow-up, appropriate interventions were delivered, and repeated surgeries were considered when necessary.

### 2.4. Evaluation of Surgical Efficacy

Surgical efficacy was evaluated by the following parameters: (1) wound healing, (2) bone defect healing rate, (3) external fixation time and external fixation index (external fixation time/length of tibial bone defects), (4) incidence or recurrence rate of deep infection, (5) postoperative complications, and (6) Association for the Study and Application of the Methods of Ilizarov (ASAMI) Score of the lower extremity [15].

## 3. Results

Infections were effectively controlled in all 68 patients with tibial bone and soft tissue defects by debridement and 1–3 times VSD or KCI treatments. The time from injury to repair and reconstruction ranged from 4 to 80 days (mean: 22 days) and the duration of postoperative follow-up from 18 to 54 months (mean: 30.80 months). Twenty-five cases were treated with local flap transfer, and all demonstrated flap survival well and wound healing. Of the 19 cases who underwent free flap transplantation to repair the wound, vascular crisis appeared in 3 cases and small area necrosis occurred after vascular exploration. The wound was healed after debridement, skin grafting, or dressing change. In patients receiving open bone transport, soft tissue defects were repaired by skin traction, but bone scars were formed. Some patients suffered from local damage due to frequent skin itching and scratching. Among them, 2 cases experienced local skin necrosis after bone grafting due to postoperative nonunion of the bone fractures. These wounds healed after dressing change for approximately one month.

The bone defects of all 68 cases were eventually reconstructed. Twelve of these cases received autogenous ilium or allogeneic bone. The mean external fixation time was 12.5 ± 3.41 months, and the mean external fixation index was 1.63 ± 0.44. Two cases receiving open bone transport required repeated resection due to exposure and infection of the transported bone segments in the process of traction. This was followed by successful bone transport without the recurrence of infection. No deep infection (osteomyelitis) occurred or recurred in the remaining patients, and no further debridement was required.

In the process of bone transport, nail canal reactions of varying severity were observed. Most of these reactions were relieved after suspending or slowing down bone transport and by dressing change. Patients with mechanical axis deviation continued bone transport after outpatient adjustment. Cases with severe nail canal reactions or mechanical axis deviation were surgically adjusted. Overall, 80 postoperative complications were encountered (Table 1). The ASAMI functional score was excellent in 34 cases (50%), good in 18 (26.47%), moderate in 10 (14.7%), and low in 6 (8.82%). Thus, 76.47% of patients achieved excellent or good results (Table 2).

### 3.1. Case 1

**Figure 1:**
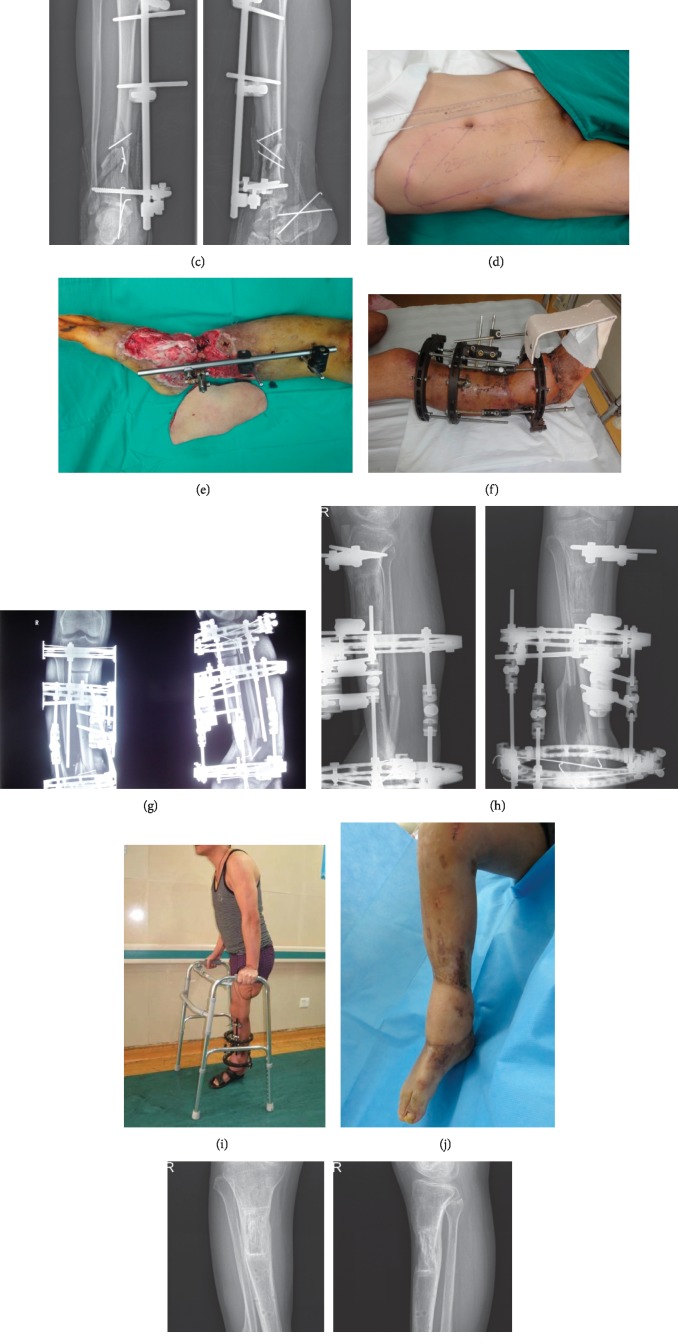
(a-b) Appearance of the affected limb when transferred to our department; (c) anteroposterior and lateral X-ray of tibia and fibula; (d-e) after debridement, limb shortening was conducted, and a thoracoumbilical flap transplanted to repair the wound surface. (f-g) 1 month after surgery, the flap survived well. The external fixator was replaced and osteotomy conducted for bone transport. Both the postoperative appearance and the X-ray result are shown; (h-i) 11 months after bone transport, the limb could bear weight, the X-ray demonstrated that the fracture was healed, and the external fixator was removed. (j-k) Both the X-ray and the appearance of the affected limb. The postoperative follow-up period was 18 months.

A 32-year-old male sustained open injury of the right lower leg (Gustilo IIIB) complicated by left lower leg destructive injury in a car accident. The middle segment of the left thigh was amputated, and the right lower leg was debrided in a local hospital. At 1.5 months after external fixation, he still had extensive tibial defect and soft tissue infection and was transferred to our department. After debridement of the right lower leg, the tibial defect measured approximately 7 cm and the wound surface was roughly 20 cm × 25 cm. The right lower leg was shortened to reduce the wound area. A thoracoumbilical flap was constructed to repair the wound. After stage-II replacement of the circular external fixator, osteotomy and bone transport were conducted, and bone and soft tissue defects healed. Postoperative external fixation time was 11 months and the external fixation index was 1.57. The final ASAMI functional score of the affected limb was graded as excellent (Figure 1).

### 3.2. Case 2

**Figure 2:**
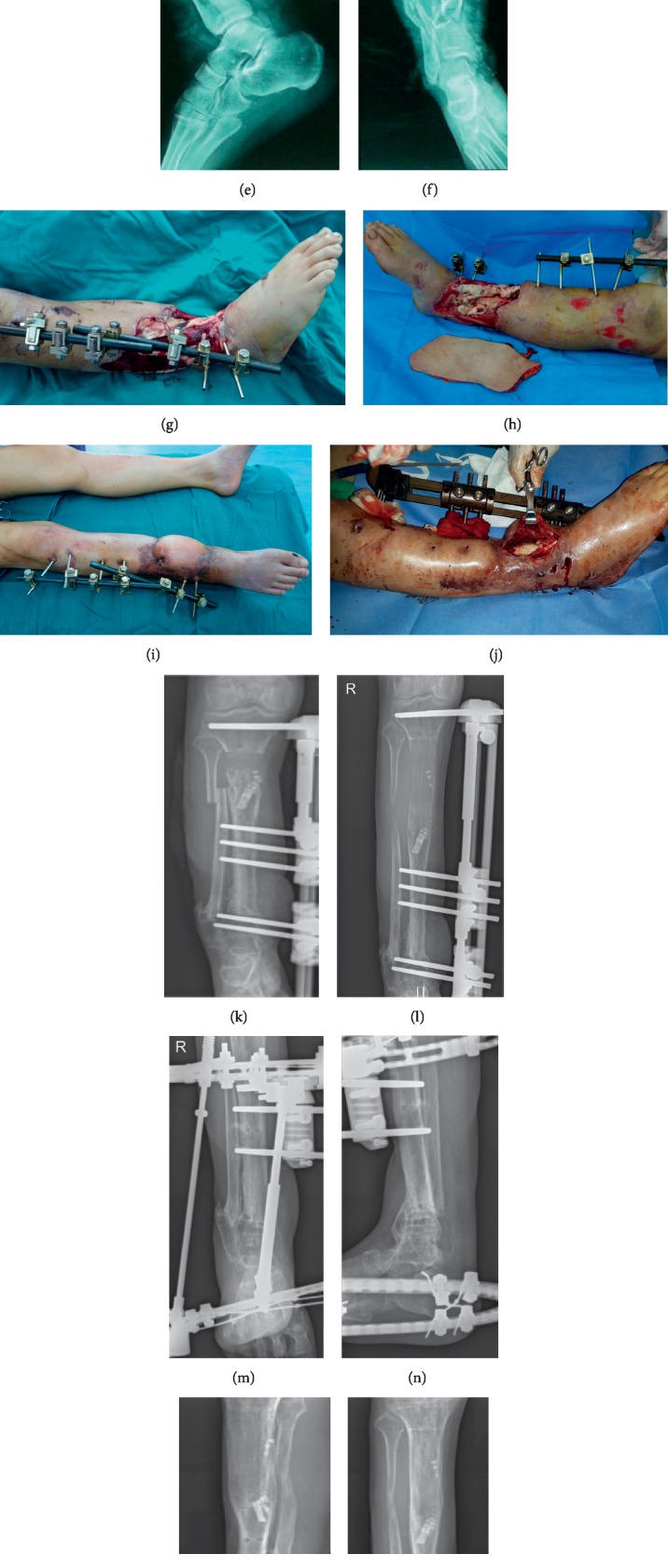
(a–f) Both the appearance and the X-rays of the injured lower leg; (g-h) free anterolateral thigh flap for repairing the wounds after multiple debridements and wound purification; (i-j) external fixator was adjusted, the lesions were debrided again, and osteotomy and bone transport were performed at the proximal tibia intraoperatively; (k-l) the X-rays of tibia and fibula during bone transport at approximately 2 months and 9 months. (m-n) Foot ring was added to correct foot drop in later stage of bone transport. 20 months after bone transport, the external fixator was scheduled to be removed. (o–r) Both the appearance and the X-ray at 36-month follow-up after the bone transport.

A 34-year-old male patient who suffered from open injuries of the right lower leg, accompanied by multiple tibial fractures and distal fibular fractures was admitted to the emergency department of our hospital after a car accident. He received repeated debridement, fracture reduction, and external fixation. During lower limb shortening, VSD of wounds was performed, and free anterolateral thigh flap was adopted to repair the wounds (approximately 12 cm × 26 cm). After 5 months of the injury, the proximal tibial bone fracture was healed, and the external fixator was adjusted. With the proximal tibial osteotomy and bone transport, the distal tibial bone defect was approximately 9 cm. Foot drop and contracture occurred during bone transport for the patient. Although foot rings were added to correct the ankle joint, the patient still suffered from the ankle joint stiffness. After 20 months of postoperative external fixation time, the ASAMI functional score of the affected limb was excellent, with an external fixation index 2.22 (Figure 2).

### 3.3. Case 3

**Figure 3:**
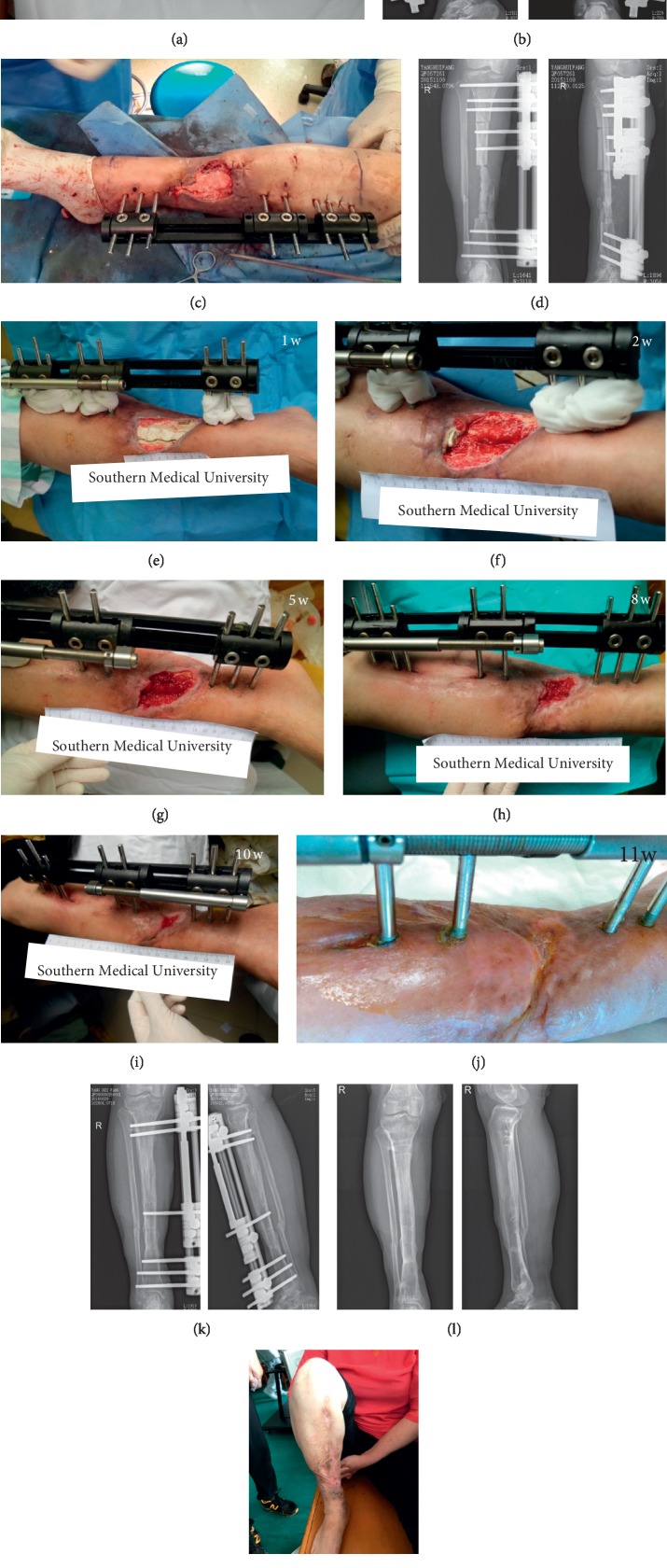
(a) The appearance of the affected limb at transfer to our department; (b) X-ray of tibia and fibula; (c) after debridement and replacement of the external fixator, the bone defect site was filled with a calcium sulfate spacer loaded with vancomycin, exposing the wound surface; (d) postoperative X-ray; (e–j) the appearance of the affected limb after operation. The wound surface gradually decreased and healed throughout the bone transport process; (k) postoperative X-ray at 11 months showed that the fracture was well healed and the external fixator was removed; (l-m) both the X-ray and the appearance at 24 months after the operation.

A 50-year-old female suffered an open injury of the right lower leg (Gustilo IIIB) due to a fall. Debridement, external fixation, free flap transplantation, and skin grafting were performed in the local hospital. She developed flap necrosis after surgery and was transferred to our department 2 months after the injury. After debridement, the tibial defect was roughly 8 cm and the wound approximately 6 cm × 8 cm. The bone and soft tissue defects healed following open bone transport. The postoperative external fixation time was 11 months and the external fixation index was 1.375. The final ASAMI functional score of the affected limb was excellent (Figure 3).

## 4. Discussion

Multiple studies have evaluated the advantages and disadvantages of the Ilizarov technique for the treatment of tibial bone defects [11, 30, 31]. Ilizarov technique is an effective treatment method for complex limb injuries which is especially applicable to developing countries or poor countries and furthermore in limited resources secondary care public hospitals [32]. Although the Ilizarov frame is not the most stable system from the biomechanical point of view it is always reliable in bone transport [33]. There are numerous challenges in treating composite tibial bone and soft tissue defects, such as sufficient soft tissue coverage, infection control, and poor bone healing during bone transport, and bone transport alone cannot fully meet therapeutic requirements. In this case series, relatively high surgical efficacy was obtained by adopting individualized treatments, including the shortening-lengthening technique, flap surgery, and open bone transport.

The shortening-lengthening technique can eliminate or reduce the distance between the two ends of the bone through temporary limb shortening, thereby facilitating early healing of the bone end and reducing the wound surface area for convenient management. Shortening-lengthening is widely applied in clinical practice, especially for cases complicated with vascular nerve defects or circular soft tissue defects [34]. If necessary, an angular deformity is slowly induced with the wound in the concavity of the defect [35]. Tetswort et al. [12] proposed that bilateral bone ends contact after acute tibial shortening. Simultaneous bone grafting is recommended to increase the healing rate. At present, there is no agreement on the maximum acute shortening length that the limb can tolerate. Sen et al. [18] recommend that the acute shortening length should not exceed 3 cm, while another study posited that the acute shortening length can be up to 6 cm for tibial defects in the middle and distal segments [36]. In the present study, the acute shortening length was determined according to the location, degree, and shape of the bone and soft tissue defects, injury time, and other specific conditions. Based on outcome success, we suggest that the shortening length can be longer than 3 cm, especially for patients with circular or semicircular soft tissue defects and deep defects. Indeed, among 47 cases receiving limb shortening, the majority were shortened by 3–5 cm (maximum: 6.5 cm) without any blood circulation disorder, and no postoperative discomfort was observed . Pierrie and Hsu [35] applied Doppler ultrasound for the recording of distal pulses to evaluate the impact of the shortening-lengthening technique on blood vessels. For cases in which could not meet the clinical need after acute shortening, an external fixator can be utilized for slow and gradual shortening [36]. Previous studies [8, 12, 37] have demonstrated that the shortening-lengthening technique requires shorter bone healing time and less frequent bone grafting compared to bone transport alone. There are, however, certain disadvantages of the shortening-lengthening technique. The fibula should be shortened simultaneously, which increases the surgical trauma. The overlapping of the fibula also increases pressure on the lower leg, which may injure the common peroneal nerve and its branches. In the process of shortening-lengthening, the loss of fibula integrity and support can lead to mechanical axis deviation of the tibia. Acute limb shortening also leads to the shortening and relaxation of muscle tendons and increases the risk of foot drop. Furthermore, most patients complain of limb pain and discomfort to varying degrees following limb shortening, probably due to vascular nerve shortening and tortuosity.

The major forms of flap surgery include the local flap transfer and the free flap transplantation. Wounds cannot be directly sutured in many patients with massive tibial bone and soft tissue defects caused by severe trauma (although the wound area can be reduced through the shortening-lengthening technique). For these cases, we recommend flap surgery for wound repair, especially for wounds adjacent to joints. In this investigation, 25 cases were treated with local flap transfer and 19 cases received free flap transfer. Although the application of flap transfer may increase surgical risk, it can cover the wound quickly, effectively control infection, and reduce the required nursing care. The flap can carry muscle fascia tissues and repair the tendon of the lower leg. Further, it can also carry cutaneous nerve or muscle branch nerves to repair the nerve deficiency of the lower leg. The flow-through vascular anastomosis technique [38] can be adopted to penetrate the vascular defects of the lower leg and improve limb blood supply. The recovery of limb function is correlated with flap coverage. Therefore, the flap should be fully utilized to repair the open wound of the deep bone, tendon, and nerve defects, thereby providing the maximum degree of tendon slip [9, 17]. The preferred donor sites of the free flap are hidden locations with constant vascular pedicle, minimal influence on function, and large resection area, such as anterolateral thigh flap, thoracoumbilical flap, latissimus dorsi flap, and lateral thoracic flap [25, 26, 39–42]. In this study, the appropriate donor site was selected according to the specific conditions of each patient, and no significant postoperative complications occurred on the donor site.

During open bone transport, soft tissue defects can heal naturally along with bone defects [37]. This technique requires neither tissue excision from other sites nor complicated surgical procedures [13, 16]. However, the open wound requires repeated dressing changes, which is a substantial inconvenience for both patient and staff, and markedly increases the incidence and recurrence rates of infection. Most soft tissue defects are irregular in shape, and the skin formed by traction may not effectively cover the wound, which is likely to increase the difficulty in the management of bone exposure. The skin migrating with bone transport is likely to sink, which affects contact and healing of bilateral ends and may require additional surgical treatment. In addition, most of the skin regenerated by traction forms scars attached to the bone, which are esthetically unsightly and likely to rupture. These problems increase the difficulties of subsequent procedures, such as the replacement of steel plates by external fixators, bone grafting, debridement of infected bone, and repair of deep tendons. Tibial bone should be removed until there is full soft tissue coverage during bone transport (i.e., there is no bone exposure at the open wound). The nail (or needle) adjacent to the defect margin should be placed as close to the defect side as possible to avoid the excessive resistance of soft tissues and loss of the traction effect. Calcium sulfate spacer insertion into the filling cavity, vacuum sealing drainage, antibacterial dressing coverage, regular dressing change, and maintaining the area dry and clean can be adopted. Robert Rozbruch et al. [37] reported a group of patients undergoing open bone transport in which the wounds of the majority were merely covered with clean gauze during bone transport, and no surgery-related infection occurred. In the current study, 13 cases underwent open bone transport. Among them, 6 cases had small wounds, which healed after soft tissue traction through bone transport for 2 weeks on average. The other seven cases were seriously injured with large wounds, but were completely healed after 1–3 months of bone transport. However, two required increased osteotomy length due to bone exposure infection. After soft tissue healing, no deep infection occurred in these 13 cases, whereas recurrent skin scratching due to pruritus was noted within 2 years after healing.

Combined with the literature [8, 9, 12, 16, 17, 35–37] and our experience, we believe that the following factors should be followed when choosing three techniques: “shortening-lengthening technique,” “flap surgery,” and “open bone transport.” (1) They are selected to be carried out according to the patient's condition, age, and willingness; (2) “Shortening-lengthening” technology is mostly applied to circular or semicircular wound, which can reduce the wound, and be combined with other two technologies; (3) “Flap surgery technology” can be considered to be applied to the larger segment of bone exposed in the wound, and the infection of exposed bone is not serious which is expected to retain more bone; the soft tissue defect involving the joint; the second stage is needed to repair tendon, nerve, and other tissue defects; (4) “Open bone transport technology” can be considered to be applied to patients with small local wound and no conditions for local flap transfer, the edge of the wound can be contacted and healed by short-term traction, or the injured area is seriously damaged and there is no usable blood vessel in the receiving area or necrosis after free flap transplantation.

Yin et al. [13] conducted a systematic review and meta-analysis of Ilizarov methods for the treatment of infectious tibia and femur nonunion including a total of 590 patients from 24 studies. Statistical analysis revealed an average tibial bone defect length of 6.54 cm, an external fixation index of 1.64 months/cm, and 1.23 complications per patient with infected tibia nonunion. In the current investigation, the methods above were adopted. The final external fixation time was 12.5 ± 3.41 and the external fixation index was 1.63 ± 0.44. The final ASAMI functional score indicated excellent/good outcome in 76.47%. The incidence of postoperative complications was 80/68 (1.18). The surgical efficacy in this study was slightly better than that reported by Yin et al. In their meta-analysis, osteomyelitis or nonunion patients without wound were enrolled, whereas all patients in this study were diagnosed with composite tibial bone and soft tissue defects, which are more difficult to repair. However, the comparison of therapeutic effects among various studies is highly subjective and the functional score after healing is significantly associated with complicated bone fractures at the adjacent site. Hutson et al. [22] and Peng et al. [14] reported that antibiotic-loaded spacers can reduce the recurrence of infection, block and reduce the invasion of surrounding soft tissues into the bone margin, and promote bone margin healing. In certain cases, antibiotic-loaded spacers (vancomycin or gentamicin) were inserted in the gaps of large bone defects. However, outcomes with and without spacers were not compared in this study.

Both severe composite tibial bone and soft tissue defects are quite challenging for orthopedic surgeons. The treatment cycle is long, the repair techniques are difficult, and both the incidences of postoperative complications and sequelae are high after wound healing. Bone transport is a kind of effective treatment for such composite injuries combined with appropriate individualized approaches (such as the shortening-lengthening technique, flap surgery, or open bone transport), which may bring the excellent surgical efficacy to the patients. However, extensive attention should be paid to the technique details throughout the treatment.

## Figures and Tables

**Table 1:** Incidence and management of postoperative complications.

Complication	Number of cases (*n* = 80)	Management	Outcome
Flap crisis	3	Vascular exploration	Slight flap necrosis was healed after debridement, grafting, or dressing change
Recurrence of deep infection	2	Removal of infected bone	Improvement
Nonunion of bone defect	14	Autologous or allogeneic bone transplantation	Healing
Refracture	4	External fixation for another 6 months	Bone union
Severe nail tunnel reaction or mechanical axis deviation	19	Nail/needle replacement, mechanical axis adjustment	Improvement
Joint stiffness (knee joint stiffness, foot drop, claw toe)	13	Decompression surgery, foot ring	Improvement
Limb shortening (>3 cm)	2	None	None
Soft tissue folding affecting bone transport contact	5	Soft tissue repair	Improvement
Flap swelling	8	Flap repair	Improvement
Poor wound healing	10	Dressing change	Improvement

Note: the number of complications refers to the number of patients presenting with complications. One patient may successively or simultaneously have different complications, and one or more may be simultaneously treated during the surgical treatment. The functional score is obtained after these complications are treated.

**Table 2:** ASAMI functional score of the lower extremity revealed excellent/good bone results in 76.47% of cases.

Functional	Description	Score
Excellent	Active, no limp, minimum stiffness (loss of <15 knee extensions/<15 dorsiflexions of ankle), no reflex sympathetic dystrophy (RSD), insignificant pain	34
Good	Active, with one or two of the following: limp, stiffness, RSD, significant pain	18
Fair	Active, with three or all of the following: limp, stiffness, RSD^a^, significant pain	10
Poor	Inactive (unemployment or inability to perform daily activities because of injury)	6
Failures	Amputation	0

## Data Availability

The data used to support the findings of this study are available from the corresponding author upon request.

## References

[1] Young K., Aquilina A., Chesser T. J. S. (2019). Open tibial fractures in major trauma centres: a national prospective cohort study of current practice. *Injury*.

[2] Toogood P., Miclau T. (2017). Critical-sized bone defects: sequence and planning. *Journal of Orthopaedic Trauma*.

[3] Grubor P., Milicevic S., Grubor M., Meccariello L. (2015). Treatment of bone defects in war wounds: retrospective study. *Medical Archives*.

[4] Bosse M. J., MacKenzie E. J., Kellam J. F. (2002). An analysis of outcomes of reconstruction or amputation after leg-threatening injuries. *New England Journal of Medicine*.

[5] Semaya A. E.-S., Badawy E., Hasan M., El-Nakeeb R. M. (2016). Management of post-traumatic bone defects of the tibia using vascularised fibular graft combined with Ilizarov external fixator. *Injury*.

[6] Ozaksar K., Sugun T. S., Toros T., Gurbuz Y., Kayalar M., Ozerkan F. (2012). Free vascularized fibular grafts in type 3 open tibia fractures. *Acta Orthopaedica et Traumatologica Turcica*.

[7] Ju J., Li L., Zhou R., Hou R. (2018). Combined application of latissimus dorsi myocutaneous flap and iliac bone flap in the treatment of chronic osteomyelitis of the lower extremity. *Journal of Orthopaedic Surgery and Research*.

[8] Wu Y., Yin Q., Rui Y., Sun Z., Gu S. (2018). Ilizarov technique: bone transport versus bone shortening-lengthening for tibial bone and soft-tissue defects. *Journal of Orthopaedic Science*.

[9] Xu J., Zhong W.-R., Cheng L. (2017). The combined use of a neurocutaneous flap and the ilizarov technique for reconstruction of large soft tissue defects and bone loss in the tibia. *Annals of Plastic Surgery*.

[10] Wang H., Wei X., Liu P. (2017). Quality of life and complications at the different stages of bone transport for treatment infected nonunion of the tibia. *Medicine*.

[11] Tong K., Zhong Z., Peng Y. (2017). Masquelet technique versus Ilizarov bone transport for reconstruction of lower extremity bone defects following posttraumatic osteomyelitis. *Injury*.

[12] Tetsworth K., Paley D., Sen C. (2017). Bone Transport versus acute shortening for the management of infected tibial non-unions with bone defects. *Injury*.

[13] Yin P., Ji Q., Li T. (2015). Systematic review and meta-analysis of ilizarov methods in the treatment of infected nonunion of tibia and femur. *PLoS One*.

[14] Peng J., Min L., Xiang Z., Huang F., Tu C., Zhang H. (2015). Ilizarov bone transport combined with antibiotic cement spacer for infected tibial nonunion. *International Journal of Clinical and Experimental Medicine*.

[15] Shahid M., Hussain A., Bridgeman P., Bose D. (2013). Clinical outcomes of the Ilizarov method after an infected tibial non union. *Archives of Trauma Research*.

[16] El-Alfy B., El-Mowafi H., El-Moghazy N. (2010). Distraction osteogenesis in management of composite bone and soft tissue defects. *International Orthopaedics*.

[17] Hollenbeck S. T., Woo S., Ong S., Fitch R. D., Erdmann D., Levin L. S. (2009). The combined use of the Ilizarov method and microsurgical techniques for limb salvage. *Annals of Plastic Surgery*.

[18] Sen C., Kocaoglu M., Eralp L., Gulsen M., Cinar M. (2004). Bifocal compression-distraction in the acute treatment of grade III open tibia fractures with bone and soft-tissue loss: a report of 24 cases. *Journal of Orthopaedic Trauma*.

[19] Paley D., Maar D. C. (2000). Ilizarov bone transport treatment for tibial defects. *Journal of Orthopaedic Trauma*.

[20] Makhdom A. M., Cartaleanu A. S., Rendon J. S., Villemure I., Hamdy R. C. (2015). The accordion maneuver: a noninvasive strategy for absent or delayed callus formation in cases of limb lengthening. *Advances in Advances in Orthopedics*.

[21] Ilizarov G. A. (1990). Clinical application of the tension-stress effect for limb lengthening. *Clinical Orthopaedics and Related Research*.

[22] Hutson J. J., Dayicioglu D., Oeltjen J. C., Panthaki Z. J., Armstrong M. B. (2010). The treatment of gustilo grade IIIB tibia fractures with application of antibiotic spacer, flap, and sequential distraction osteogenesis. *Annals of plastic surgery*.

[23] Calori G. M., Mazza E., Colombo M., Ripamonti C. (2011). The use of bone-graft substitutes in large bone defects: any specific needs?. *Injury*.

[24] Bao T., Han F., Xu F. (2017). Papineau technique combined with vacuum-assisted closure for open tibial fractures: clinical outcomes at five years. *International Orthopaedics*.

[25] Li R.-G., Yu B., Wang G. (2012). Sequential therapy of vacuum sealing drainage and free-flap transplantation for children with extensive soft-tissue defects below the knee in the extremities. *Injury*.

[26] Li R. G., Ren G. H., Tan X. J., Yu B., Hu J. J. (2013). Free flap transplantation combined with skin grafting and vacuum sealing drainage for repair of circumferential or sub-circumferential soft-tissue wounds of the lower leg. *Medical Science Monitor*.

[27] Gao-Hong R., Run-Guang L., Gui-Yong J., Chao-Jie C., Zhi-Gang B. (2017). A solution to the vessel shortage during free vascularized fibular grafting for reconstructing infected bone defects of the femur: bridging with vein transplantation. *Injury*.

[28] Bisaccia M., Rinonapoli G., Meccariello L., Caraffa A., Cukierman B., Iborra J. R. (2017). The challenges of monoaxial bone transport in orthopedics and traumatology. *Ortopedia, Traumatologia, Rehabilitacja*.

[29] Nayagam S. (2007). Safe corridors in external fixation: the lower leg (tibia, fibula, hindfoot and forefoot). *Strategies in Trauma and Limb Reconstruction*.

[30] Abdelkhalek M., El-Alfy B., Ali A. M. (2016). Ilizarov bone transport versus fibular graft for reconstruction of tibial bone defects in children. *Journal of Pediatric Orthopaedics B*.

[31] El-Gammal T. A., Shiha A. E., El-Deen M. A. (2008). Management of traumatic tibial defects using free vascularized fibula or Ilizarov bone transport: a comparative study. *Microsurgery*.

[32] Khan M. S., Giacomo L., Bisaccia M. (2018). Ilizarov technique, satisfactory outcome with limited resources. *Clinical Cases in Mineral and Bone Metabolism*.

[33] Grubor P., Mitkovi M., Grubor M., Mitkovi M., Meccariello L., Falzarano A. (2016). Biomechanical stability of juvidur and bone models on osteosyntesic materials. *Acta Informatica Medica*.

[34] Fletcher M. D. A., Solomin L. N. (2015). Definitive management of significant soft tissue loss associated with open diaphyseal fractures utilising circular external fixation without free tissue transfer, a comprehensive review of the literature and illustrative case. *European Journal of Orthopaedic Surgery & Traumatology*.

[35] Pierrie S. N., Hsu J. R. (2017). Shortening and angulation strategies to address composite bone and soft tissue defects. *Journal of Orthopaedic Trauma*.

[36] El-Rosasy M. A. (2007). Acute shortening and re-lengthening in the management of bone and soft-tissue loss in complicated fractures of the tibia. *The Journal of Bone and Joint Surgery. British volume*.

[37] Robert Rozbruch S., Weitzman A. M., Tracey Watson J., Freudigman P., Katz H. V., Ilizarov S. (2006). Simultaneous treatment of tibial bone and soft-tissue defects with the Ilizarov method. *Journal of Orthopaedic Trauma*.

[38] Li Z., Yu A., Qi B., Pan Z., Ding J. (2017). Flow-through free fibula osteocutaneous flap in reconstruction of tibial bone, soft tissue, and main artery segmental defects. *Annals of Plastic Surgery*.

[39] Korompilias A. V., Lykissas M. G., Vekris M. D., Beris A. E., Soucacos P. N. (2008). Microsurgery for lower extremity injuries. *Injury*.

[40] Sekido M., Yamamoto Y., Furukawa H., Sugihara T. (2004). Change of weight-bearing pattern before and after plantar reconstruction with free anterolateral thigh flap. *Microsurgery*.

[41] Hwang K. T., Kim S. W., Sung I. H., Kim J. T., Kim Y. H. (2016). Is delayed reconstruction using the latissimus dorsi free flap a worthy option in the management of open IIIB tibial fractures?. *Microsurgery*.

[42] Repo J. P., Barner-Rasmussen I., Roine R. P., Sintonen H., Tukiainen E. J. (2016). Treatment of compound tibia fracture with microvascular latissimus dorsi flap and the Ilizarov technique: a cross-sectional study of long-term outcomes. *Journal of Plastic, Reconstructive & Aesthetic Surgery*.

